# Performance of the comprehensive nutrition screening index in predicting mortality after cardiac surgery

**DOI:** 10.1038/s41598-024-78114-x

**Published:** 2024-11-18

**Authors:** Jaeyeon Chung, Jinyoung Bae, Seyong Park, Dong Hyouk Kim, Youn Joung Cho, Karam Nam, Yunseok Jeon, Jae-Woo Ju

**Affiliations:** 1 Department of Anesthesiology and Pain Medicine, Seoul National University Hospital, Seoul National University College of Medicine, Seoul, Republic of Korea; 2 Department of Anesthesiology and Pain Medicine, Ajou University Medical Center, Ajou University School of Medicine, Suwon, Republic of Korea

**Keywords:** Cardiac surgery, Preoperative malnutrition, Screening, SNUH-NSI (Seoul National University Hospital-Nutrition Screening Index), Mortality, Medical research, Malnutrition

## Abstract

Malnutrition is a significant risk factor for postoperative outcomes in patients undergoing cardiac surgery. The Seoul National University Hospital-Nutrition Screening Index (SNUH-NSI) is an automated screening tool designed to comprehensively assess malnutrition risk. However, its predictive value in patients undergoing cardiac surgery remains unvalidated. This study included adult patients who underwent cardiac surgery at a tertiary teaching hospital between May 2008 and December 2019. Patients were classified as low-, intermediate-, or high-risk for malnutrition based on the SNUH-NSI. The association between malnutrition risk and postoperative cumulative all-cause mortality was evaluated using multivariable Cox regression analysis. The predictive ability of the SNUH-NSI was compared with conventional nutritional indices. Compared to the low-risk group, both the high-risk (adjusted hazard ratio [HR], 2.64; 95% confidence interval [CI], 2.19–3.19; *P* < 0.001) and intermediate-risk groups (adjusted HR, 1.54; 95% CI, 1.31–1.82; *P* < 0.001) demonstrated a significantly elevated risk of cumulative all-cause mortality. The multivariable model incorporating SNUH-NSI demonstrated moderate but superior predictive performance compared to that of other conventional nutritional indices. Preoperative malnutrition assessed by the SNUH-NSI was found to be a significant predictor of mortality following cardiac surgery. Implementing the SNUH-NSI for automated preoperative malnutrition screening could be an efficient and cost-effective approach.

**Supplementary Information:**

The online version contains supplementary material available at 10.1038/s41598-024-78114-x.

## Introduction

Patients undergoing cardiac surgery frequently present with preoperative malnutrition, with a prevalence reaching 20%^1,2^. Surgical trauma induces a stress response with hormonal and inflammatory mediators crucial for the maintenance of metabolic and physiological homeostasis, which are responsible for body healing and immune response^3,4^. However, malnutrition can prolong this response and consequently delay postoperative recovery^5,6^. Specifically, in cardiac surgery, preoperative malnutrition may exacerbate the inflammatory response caused by cardiopulmonary bypass, prolong hospitalization, and increase the risk of postoperative infections, delirium, and mortality^7–10^. As malnutrition is considered one of the few modifiable risk factors associated with postoperative outcomes^11^, current guidelines recommend routine preoperative nutritional status screening to identify those needing nutritional optimization^12^. In fact, the enhanced recovery after surgery protocol recognizes the importance of preoperative nutritional screening and subsequent interventions to improve post-cardiac surgery outcomes^13,14^. Despite the availability of various preoperative nutritional screening tools, certain limitations are present due to the reliance on few parameters, sole use of subjective patient responses, or need for extensive assessment^7,15^.

The Seoul National University Hospital-Nutrition Screening Index (SNUH-NSI) is a comprehensive institutional screening tool for assessing malnutrition risk using 11 routinely obtained laboratory and clinical parameters during the preoperative period. This allows for the automated evaluation of malnutrition risk in all patients undergoing surgery. Previous studies have reported its effectiveness in predicting postoperative complications and in-hospital mortality following abdominal surgery^16–18^. However, evidence on its utility in predicting outcomes for patients undergoing cardiac surgery is limited.

Therefore, this retrospective study aimed to evaluate the association between malnutrition risk based on the SNUH-NSI and postoperative mortality in patients undergoing cardiac surgery. The predictive value of SNUH-NSI for postoperative mortality was also compared with conventional nutritional indices.

## Methods

### Study population

This retrospective observational study included adult patients who underwent cardiac surgery at a tertiary teaching hospital. The study protocol was reviewed and approved by the Institutional Review Board of our institution of Seoul National University Hospital (approval no. H-2106-009-1223) on June 4, 2021. Owing to the de-identified nature of the data, the need for written informed consent was waived by the Institutional Review Board of Seoul National University Hospital. The study adhered to the ethical principles of the 2013 Declaration of Helsinki and the Strengthening the Reporting of Observational Studies in Epidemiology guidelines.

Electronic medical records of adult patients (≥ 19 years of age) who underwent coronary artery bypass grafting surgery (CABG), heart valve surgery, and thoracic aorta surgery at a tertiary teaching hospital between May 2008 and December 2019 were retrospectively reviewed. Patients who underwent repeated cardiac surgery during the study period, those with missing covariate values, and those who were not assessed with SNUH-NSI prior to the index surgery were excluded from the analysis.

### SNUH-NSI

The definition and institutional protocol for SNUH-NSI have been used at our institution since 2010. For all inpatients expected to stay at the hospital for at least one night, the SNUH-NSI was used to routinely assess malnutrition risk within 24 h of admission. This process was automated, and the results were documented in the electronic medical records. The SNUH-NSI incorporates 11 parameters that are routinely obtained before surgery, including six clinical parameters (appetite, weight change, digestion difficulties, diet type, body mass index [BMI], and age) and five laboratory parameters (serum albumin level, serum cholesterol level, total lymphocyte count, serum hemoglobin level, and C-reactive protein) (Table 1). Clinical parameters are regularly recorded during admission in nursing records, and all laboratory parameters are part of routine preoperative panels. Each parameter is categorized as R1 (high risk), R2 (intermediate risk), or R3 (low risk) based on its association with malnutrition severity. Missing parameters at the time of assessment were considered as R3. Based on these parameters, nutritional status was classified into three groups (high, intermediate, and low risk of malnutrition). The high-risk group included patients with more than two R1 parameters, or one R1 and more than two R2 parameters. The intermediate-risk group included patients with one R1 or more than two R2 parameters. Lastly, the low-risk group included patients with one R2 or all R3 parameters (Table 1).

**Table 1:** Seoul National University Hospital-Nutrition Screening Index.

	R1	R2	R3
Appetite	Bad	–	Normal/good
Change of weight	Yes	–	No
Difficulty in digesting	–	Yes	No
Diet type	Fluid diet	Soft blended diet or NPO	Normal regular diet
Serum albumin (g/dL)	< 2.8	2.8–3.3	≥ 3.3
Serum cholesterol (mg/dL)	–	< 130	≥ 130
Total lymphocyte count (cells/mm^3^)	< 800	800–1,500	≥ 1,500
Hemoglobin (g/dL)	–	Male < 13.0 Female < 12.0	Male ≥ 13.0 Female ≥ 12.0
C-reactive protein (mg/dL)	–	> 1	≤ 1
Body mass index (kg/m^2^)	–	< 18 or ≥ 25	18–25
Age (years)	–	> 75	≤ 75

R, risk; NPO, nil per os.

High risk group, (more than 2 parameters under R1) or (1 parameter under R1 and more than 2 parameters under R2); intermediate risk group, (1 parameter under R1) or (more than 2 parameters under R2); low risk group, (only 1 parameter under R2) or (all parameters under R3).

### Data collection

Electronic medical records were reviewed using the database system of our institution. The following variables were recorded: SNUH-NSI and its component parameters; smoking; hazardous alcohol consumption (defined as an alcohol consumption 36 g of ethanol/day)^19^; sex; past medical history (hypertension, diabetes mellitus, dyslipidemia, atrial fibrillation or flutter, myocardial infarction, congestive heart failure, stroke or transient ischemic attack, chronic obstructive pulmonary disease, extracardiac arteriopathy, chronic kidney disease, and dialysis); previous cardiac surgery; preoperative laboratory data (serum creatinine [mg/dL] and left ventricular ejection fraction [%]); type of surgery (CABG, valve, aorta, valve + CABG, and valve + aorta); emergency surgery; year of surgery (2008–2011, 2012–2015, and 2016–2019); duration of surgery (min); and amount of packed red blood cell transfusion (units).

Data measured from conventional nutritional indices and their associated parameters were also collected. Serum albumin levels were classified into two groups, with cut-off values of 2.5 g/dL^20^ and 3.0 g/dL^21^. BMI was classified based on the World Health Organization classification (BMI WHO; underweight, < 18.5 kg/m^2^; normal, 18.5–24.9 kg/m^2^; overweight, 25–29.9 kg/m; obese, ≥ 30 kg/m^2^) and Asia-Pacific classification (BMI Asia; underweight, < 18.5 kg/m^2^; normal, 18.5–22.9 kg/m^2^; overweight, 23–24.9 kg/m^2^; obese, ≥ 25 kg/m^2^)^22^. The Nutritional Risk Index (NRI) was calculated using the following formula: 1.519 × serum albumin (g/dL) + [41.7 × weight (kg) / ideal body weight (kg)]. NRI values were then classified as having no risk (≥ 100), mild risk (97.5–100), moderate risk (83.5–97.5), and severe risk (< 83.5) of malnourishment^23,24^. The chart-derived frailty index (CFI) was calculated as the sum of the following parameters: age > 70 years, BMI < 18.5 kg/m^2^, serum hematocrit < 35%, serum albumin < 3.4 g/dL, or serum creatinine > 2.0 mg/dL^25^. CFI values were then classified as having a low (0–2) or high (3–5) frailty risk^25^.

### Study outcomes

The primary outcome was the risk of cumulative all-cause mortality according to the SNUH-NSI. Secondary outcomes included cumulative cardiac-cause and in-hospital mortalities. Mortality data were obtained from electronic medical records and the National Population Registry of the Korean National Statistical Office. Follow-up for cumulative all-cause mortality was uniformly censored on December 31, 2019, or hospital discharge, whichever occurred later. Cardiac-cause mortality was defined as death from myocardial infarction, fatal arrhythmia, heart failure, pulmonary embolism, or cardiogenic shock. The primary cause of in-hospital deaths was also collected by reviewing the electronic medical records. The other outcomes including acute kidney injury (defined as per the serum creatinine criteria of the Kidney Disease: Improving Global Outcomes definition)^26^, newly initiated renal replacement therapy within 7 days of surgery, prolonged intubation (defined as cases where tracheal intubation was required beyond 48 h postoperatively), length of stay in the intensive care unit and hospital, and readmission within 90 days of discharge, were also collected.

### Statistical analysis

Continuous variables were expressed as medians with interquartile ranges (IQRs), whereas categorical variables were expressed as frequencies and percentages. The Kruskal-Wallis test was used for comparisons of continuous variables, while the χ^2^ test or Fisher’s exact test was used for categorical variables. Statistical analyses were performed using the Statistical Package for Social Science (SPSS; version 22.0; IBM Corp., Armonk, NY, USA; https://www.ibm.com/products/spss-statistics) or R software (version 4.3.0; R Foundation for Statistical Computing, Vienna, Austria; https://www.r-project.org), and statistical significance was set at *P* < 0.05.

As a preliminary analysis, the association between each SNUH-NSI parameter and the risk of all-cause mortality was evaluated using univariable Cox proportional hazards regression analysis. Then, to investigate the association between SNUH-NSI and the risk of cumulative all-cause mortality following cardiac surgery, Kaplan-Meier analysis and log-rank tests were employed. Multivariable Cox proportional hazards regression analyses were also performed to estimate hazard ratios (HRs) and 95% confidence intervals (CIs). Univariable Cox regression analyses were performed for the SNUH-NSI and established risk factors known for postoperative outcomes after cardiac surgery^27^: age; sex; smoking; hazardous alcohol consumption; past medical history (diabetes mellitus, myocardial infarction, congestive heart failure, stroke/transient ischemic attack, chronic obstructive pulmonary disease, extracardiac arteriopathy, and dialysis); previous cardiac surgery; serum creatinine level; left ventricular ejection fraction; type of surgery; year of surgery; and emergency surgery status. While age was already included as a parameter in the SNUH-NSI, it was also included as a covariate owing to its significant impact on cumulative mortality. Chronic kidney disease was excluded to avoid multicollinearity with history of dialysis and serum creatinine levels. Subsequently, variables with a *P* < 0.1 were entered into a multivariable Cox regression model without applying further selection methods. Proportional hazard assumptions were tested using a log-log plot. The performance of the multivariable Cox regression model incorporating SNUH-NSI in predicting cumulative all-cause mortality was evaluated using Harrell’s C-index (C-index), Heagerty’s integrated area under the receiver operating curve (iAUC) at the 1-year time point, and Cox and Snell’s pseudo R^2^. These measures were then compared to other multivariable Cox regression models, each containing the same covariates but with a different alternative nutritional index. The risk of cumulative cardiac-cause mortality was similarly evaluated using the same methods.

To assess the association between the SNUH-NSI and in-hospital mortality following cardiac surgery, logistic regression analyses were performed to estimate odds ratios (ORs) and 95% CIs. Univariable and multivariable analyses were conducted similarly to the Cox regression analyses. The performance of the multivariable logistic regression model incorporating the SNUH-NSI in predicting in-hospital mortality was evaluated using the C-index, area under the receiver operating curve (AUC), Akaike information criterion (AIC), and McFadden’s pseudo R^2^. These measures were subsequently compared among different multivariable logistic regression models, similar to the approach used for comparing the Cox regression models.

To ensure the robustness of our findings, we performed a sensitivity analysis to examine whether treating the missing parameters for the SNUH-NSI as R3 (low-risk) might have affected our results. For patients with complete data on the SNUH-NSI parameters, log-rank test and multivariable Cox proportional hazards regression analyses were repeated for the primary outcome.

## Results

**Fig. 1:**
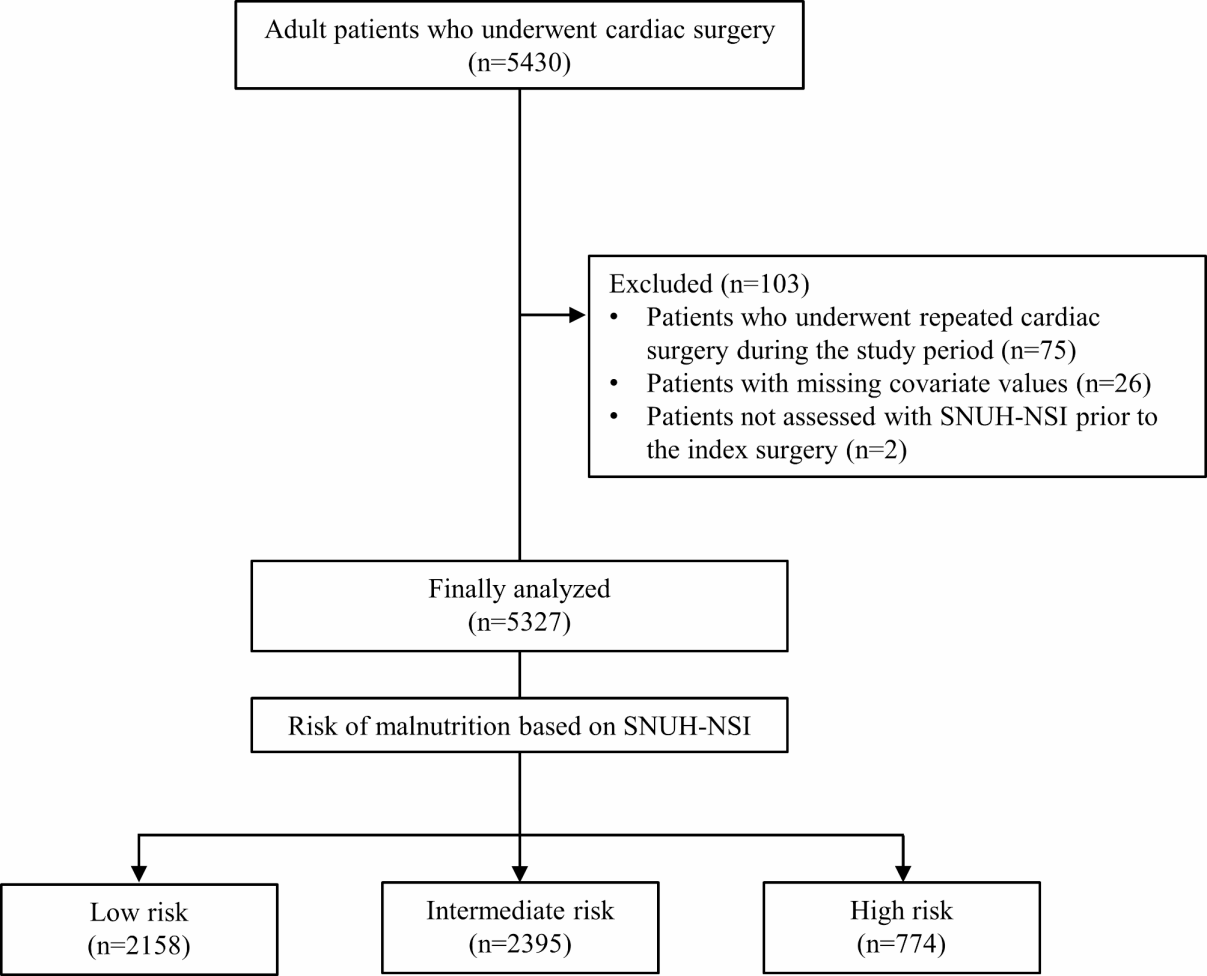
Flowchart of the study.

Among the 5,430 patients who met the inclusion criteria, 75 were excluded owing to repeat cardiac surgery during the study period, 26 owing to missing covariate values, and two owing to absent preoperative SNUH-NSI assessment. Ultimately, 5,327 patients were included in the final analysis (Fig. 1). Baseline characteristics and perioperative data are presented in Table 2. Based on the SNUH-NSI assessment, 2,158, 2,395, and 774 patients were grouped into the low-, intermediate-, and high-risk groups, respectively. The median (IQR) duration from admission to the day of surgery was 5 (3–7) days.

**Table 2:** Patient characteristics and perioperative data according to the study groups.

	SNUH-NSI			
	Low risk (*n* = 2,158)	Intermediate risk (*n* = 2,395)	High risk (*n* = 774)	*P* value
SNUH-NSI parameters				
Appetite^a^	0 (0)	40 (1.7)	236 (30.5)	< 0.001
Change of weight^b^	0 (0)	203 (8.5)	396 (51.2)	< 0.001
Difficulty in digesting^c^	35 (1.6)	169 (7.1)	139 (18.0)	< 0.001
Diet type^d^				< 0.001
Normal regular diet	2,108 (97.7)	2,013 (84.1)	549 (70.9)	
Soft blended diet or NPO	50 (2.3)	381 (15.9)	205 (26.5)	
Fluid diet	0 (0)	1 (0.0)	20 (2.6)	
Serum albumin (g/dL)	4.2 (4.0–4.4)	3.9 (3.6–4.2)	3.5 (3.0–4.0)	< 0.001
Serum cholesterol (mg/dL)^e^	168 (147–194)	146 (122–174)	136 (114–163)	< 0.001
Total lymphocyte count (cells/mm^3^)^f^	2,020 (1,709–2,471)	1,555(1,230–2,094)	1,103 (712–1,646)	< 0.001
Hemoglobin (g/dL)	13.7 (12.9–14.7)	12.4 (11.0–13.6)	11.2 (9.9–12.6)	< 0.001
C-reactive protein (mg/dL)^g^	0.1 (0.0–0.2)	0.2 (0.1–0.7)	0.6 (0.1–4.0)	< 0.001
Body mass index (kg/m^2^)	23.6 (21.7–25.3)	23.8 (21.0–26.2)	21.9 (18.6–25.0)	< 0.001
Age (years)	60 (52–68)	68 (59–74)	67 (56–74)	< 0.001
Male	1,266 (58.7)	1,495 (62.4)	459 (59.3)	0.027
Smoker	348 (16.1)	347 (14.5)	125 (16.1)	0.255
Hazardous alcohol consumption	64 (3.0)	66 (2.8)	35 (4.5)	0.043
Comorbidities				
Hypertension	884 (41.0)	1,330 (55.5)	381 (49.2)	< 0.001
Diabetes mellitus	394 (18.3)	762 (31.8)	234 (30.2)	< 0.001
Dyslipidemia	363 (16.8)	476 (19.9)	93 (12.0)	< 0.001
Atrial fibrillation/flutter	364 (16.9)	435 (18.2)	208 (26.9)	< 0.001
Myocardial infarction	71 (3.3)	177 (7.4)	49 (6.3)	< 0.001
Congestive heart failure	101 (4.7)	229 (9.6)	159 (20.5)	< 0.001
Stroke/transient ischemic attack	240 (11.1)	387 (16.2)	110 (14.2)	< 0.001
Chronic obstructive pulmonary disease	35 (1.6)	39 (1.6)	19 (2.5)	0.265
Extracardiac arteriopathy	53 (2.5)	112 (4.7)	29 (3.7)	< 0.001
Chronic kidney disease	459 (21.3)	650 (27.1)	223 (28.8)	< 0.001
Dialysis	20 (0.9)	87 (3.6)	45 (5.8)	< 0.001
Previous cardiac surgery	120 (5.6)	190 (7.9)	99 (12.8)	< 0.001
Preoperative laboratory data				
Serum creatinine (mg/dl)	0.9 (0.8–1.0)	0.9 (0.8–1.2)	1.0 (0.8–1.3)	< 0.001
Left ventricular ejection fraction (%)	59 (54–64)	58 (51–63)	56 (45–62)	< 0.001
Type of surgery				< 0.001
CABG	644 (29.8)	1,003 (41.9)	190 (24.5)	
Valve	852 (39.5)	784 (32.7)	340 (43.9)	
Aorta	120 (5.6)	172 (7.2)	66 (8.5)	
Valve + CABG	459 (21.3)	375 (15.7)	150 (19.4)	
Valve + aorta	83 (3.8)	61 (2.5)	28 (3.6)	
Emergency surgery	173 (8.0)	320 (13.4)	181 (23.4)	< 0.001
Year of surgery				0.008
2008–2011	700 (32.4)	732 (30.6)	234 (30.2)	
2012–2015	680 (31.5)	811 (33.9)	298 (38.5)	
2016–2019	778 (36.1)	852 (35.6)	242 (31.3)	
Duration of surgery (minutes)	415 (350–475)	435 (375–500)	460 (385–550)	< 0.001
Packed red blood cell transfusion (units)	0 (0–1)	1 (0–3)	1 (0–3)	< 0.001

SNUH-NSI, Seoul National University Hospital-Nutrition Screening Index; NPO, nil per os; CABG, coronary artery bypass grafting surgery; ICU, intensive care unit;

Values are expressed as the median (interquartile range) or number (proportion).

^a^ 71 missing values.

^b^ 120 missing values.

^c^ 9 missing values.

^d^ 29 missing values.

^e^ 46 missing values.

^f^ 389 missing values.

^g^ 61 missing values.

### Primary outcome

**Fig. 2:**
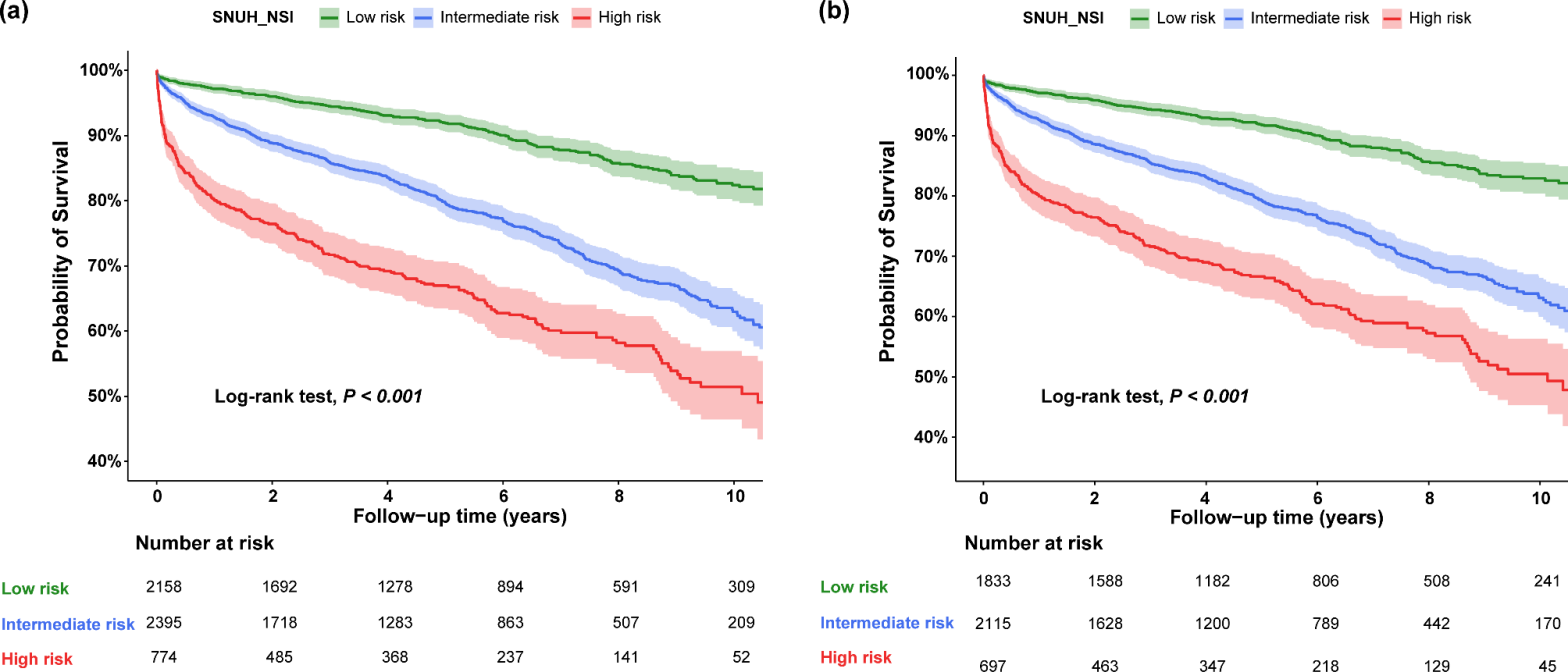
Kaplan–Meier curve for all-cause mortality classified by Seoul National University Hospital-Nutrition Screening Index (SNUH-NSI) in (**a**) the total cohort and (**b**) patients with complete data on the SNUH-NSI.

During the median (IQR) follow-up duration of 4.5 (1.8–7.7) years, the overall cumulative all-cause mortality rate was 19.4% (1,036/5,327). Specifically, mortality rates of 10.2% (220/2,158), 22.7% (543/2,395), and 35.3% (273/774) were observed in the low-, intermediate-, and high-risk groups, respectively. All SNUH-NSI parameters except BMI were significantly associated with cumulative all-cause mortality (Supplemental Table **S1**). The Kaplan-Meier curve for cumulative all-cause mortality is shown in Fig. 2a. Significant differences in cumulative all-cause mortality were observed across the study groups, with the highest mortality in the high-risk group and the lowest in the low-risk group (log-rank test, pairwise comparison; all *P* < 0.001).

The results of the univariable and multivariable Cox regression analyses for all-cause mortality are presented in Table 3. The multivariable analysis determined the intermediate-risk (adjusted HR, 1.54; 95% CI, 1.31–1.82; *P* < 0.001) and the high-risk groups (adjusted HR 2.64; 95% CI, 2.19–3.19; *P* < 0.001) as significant risk factors for cumulative all-cause mortality compared with the low-risk group. The predictive performance of the multivariable Cox regression models for cumulative all-cause mortality is presented in Table 4. The multivariable model incorporating SNUH-NSI exhibited the highest iAUC (0.743). Additionally, the C-index (0.750) and pseudo R^2^ (0.575) values of the SNUH-NSI surpassed that of other models, except for the NRI model (C-index, 0.751; pseudo R^2^, 0.578; Table 4).

**Table 3:** Univariable and multivariable Cox regression analysis for all-cause mortality after cardiac surgery.

	Univariable		Multivariable	
	Unadjusted HR (95% CI)	*P* value	Adjusted HR (95% CI)	*P* value
SNUH-NSI		< 0.001		
Low-risk	Reference		Reference	
Intermediate-risk	2.48 (2.12–2.90)	< 0.001	1.54 (1.31–1.82)	< 0.001
High-risk	4.38 (3.67–5.24)	< 0.001	2.64 (2.19–3.19)	< 0.001
Age (years)	1.06 (1.06–1.07)	< 0.001	1.06 (1.05–1.07)	< 0.001
Male	1.27 (1.11–1.44)	< 0.001	1.21 (1.05–1.38)	0.006
Smoker	1.00 (0.85–1.18)	0.974		
Hazardous alcohol consumption	0.94 (0.66–1.35)	0.748		
Comorbidities				
Diabetes mellitus	1.71 (1.51–1.94)	< 0.001	1.16 (1.01–1.33)	0.037
Myocardial infarction	1.52 (1.19–1.93)	< 0.001	0.98 (0.76–1.26)	0.878
Congestive heart failure	1.96 (1.66–2.32)	< 0.001	1.21 (1.00–1.46)	0.046
Stroke/transient ischemic attack	1.63 (1.38–1.91)	< 0.001	1.24 (1.05–1.46)	0.012
Chronic obstructive pulmonary disease	2.00 (1.35–2.98)	< 0.001	1.16 (0.77–1.73)	0.477
Extracardiac arteriopathy	1.85 (1.43–2.41)	< 0.001	1.25 (0.95–1.63)	0.109
Dialysis	5.01 (4.01–6.26)	< 0.001	2.12 (1.49–3.02)	< 0.001
Previous cardiac surgery	1.20 (0.96–1.49)	0.111		
Preoperative laboratory data				
Serum creatinine (mg/dL)	1.23 (1.20–1.26)	< 0.001	1.10 (1.06–1.15)	< 0.001
Left ventricular ejection fraction (%)	0.98 (0.97–0.98)	< 0.001	0.98 (0.98–0.99)	< 0.001
Type of surgery		< 0.001		
CABG	Reference		Reference	
Valve	0.73 (0.63–0.85)	< 0.001	1.14 (0.97–1.34)	0.112
Aorta	1.10 (0.87–1.38)	0.443	1.48 (1.16–1.89)	0.002
Valve + CABG	0.77 (0.65–0.92)	0.003	1.13 (0.94–1.36)	0.195
Valve + aorta	0.64 (0.34–1.20)	0.166	1.19 (0.63–2.25)	0.588
Year of surgery		0.108		
2008–2011	Reference			
2012–2015	1.05 (0.91–1.22)	0.476		
2016–2019	0.86 (0.70–1.04)	0.120		
Emergency surgery	1.46 (1.25–1.70)	< 0.001	1.04 (0.88–1.22)	0.657

HR, hazard ratio; CI, confidence interval; SNUH-NSI, Seoul National University Hospital-Nutrition Screening Index; CABG, coronary artery bypass grafting.

**Table 4:** Performance of Cox regression models for all-cause mortality based on different nutrition indexes.

Nutrition index		Harrell’s C-index	iAUC	Cox & Snell pseudo *R*^2^
SNUH-NSI	Low vs. Intermediate vs. High	0.750	0.743	0.575
Serum albumin	< 2.5 g/dL vs. ≥2.5 g/dL	0.737	0.729	0.537
	< 3.0 g/dL vs. ≥3.0 g/dL	0.740	0.731	0.545
BMI WHO (kg/m^2^)	< 18.5 vs. 18.5 ≤ BMI < 25 vs. 25 ≤ BMI < 30 vs. ≥30	0.741	0.732	0.548
BMI Asia (kg/m^2^)	< 18.5 vs. 18.5 ≤ BMI < 23 vs. 23 ≤ BMI < 25 vs. ≥25	0.741	0.733	0.551
Nutritional Risk Index	> 100 (no risk) vs. 97.5 ≤ NRI < 100 (mild) vs. 83.5 ≤ NRI < 97.5 (intermediate) vs. ≤83.5 (severe)	0.751	0.740	0.578
Chart-derived Frailty Index	0–2 (low risk) vs. 3–5 (high risk)	0.741	0.731	0.555
Multivariable model without Nutrition index	–	0.734	0.728	0.532

iAUC, integrated area under the receiver operating curve; SNUH-NSI, Seoul National University Hospital-Nutrition Screening Index; BMI, Body mass index; WHO, World Health Organization classification; Asia, Asia-Pacific classification.

### Secondary outcomes

The cumulative incidence of cardiac-cause mortality was 5.8% (312/5,327). The results of univariable and multivariable Cox regression analyses for cardiac-cause mortality are summarized in **Supplemental Table S2**. Multivariable Cox regression analysis revealed a higher risk of cardiac-cause mortality in the moderate-risk group (adjusted HR, 1.39; 95% CI, 1.02–1.89; *P* = 0.036) and the high-risk group (adjusted HR, 2.68; 95% CI, 1.91–3.77; *P* < 0.001) than that in the low-risk group (**Supplemental Table S2**). Among the various multivariable models for cardiac-cause mortality, the model incorporating the SNUH-NSI demonstrated the highest predictive performance (C-index, 0.773; iAUC, 0.767; pseudo R^2^, 0.659; Table 5).

**Table 5:** Performance of multivariable Cox regression models for cardiac-cause mortality based on different nutrition indexes.

Nutrition index		C-index	iAUC	Cox & Snell pseudo *R*^2^
SNUH-NSI	Low vs. Intermediate vs. High	0.773	0.767	0.659
Serum albumin	< 2.5 g/dL vs. ≥2.5 g/dL	0.762	0.756	0.622
	< 3.0 g/dL vs. ≥3.0 g/dL	0.762	0.757	0.624
BMI WHO (kg/m^2^)	< 18.5 vs. 18.5 ≤ BMI < 25 vs. 25 ≤ BMI < 30 vs. ≥30	0.763	0.757	0.626
BMI Asia (kg/m^2^)	< 18.5 vs. 18.5 ≤ BMI < 23 vs. 23 ≤ BMI < 25 vs. ≥25	0.763	0.758	0.629
Nutritional Risk Index	> 100 (no risk) vs. 97.5 ≤ NRI < 100 (mild) vs. 83.5 ≤ NRI < 97.5 (intermediate) vs. ≤83.5 (severe)	0.772	0.762	0.646
Chart-derived Frailty Index	0–2 (low risk) vs. 3–5 (high risk)	0.763	0.755	0.630
Model without Nutrition index	–	0.760	0.755	0.618

C-index, Harrell’s C-index; iAUC, integrated area under the receiver operating curve; SNUH-NSI, Seoul National University Hospital-Nutrition Screening Index; BMI, Body mass index; WHO, World Health Organization classification; Asia, Asia-Pacific classification.

The in-hospital mortality rate was 2.6% (137/5,327 patients). The causes of in-hospital mortality are described in **Supplemental Table S3**. In multivariable logistic regression analysis, the risk of in-hospital mortality was significantly higher in the high-risk group (adjusted OR, 3.14; 95% CI, 1.87–5.26; *P* < 0.001) than in the low-risk group (**Supplemental Table S4**). However, the intermediate-risk group showed no significant differences in in-hospital mortality compared to other groups (adjusted OR, 1.19; 95% CI, 0.71–1.97; *P* = 0.510, **Supplemental Table S4**). Among the various multivariable logistic regression models for in-hospital mortality, the model incorporating the SNUH-NSI exhibited the highest predictive performance (AUC [95% CI], 0.831 (0.804–0.866); pseudo R^2^, 0.175; AIC, 1082.5; **Supplemental Table S5**,** Supplemental Fig. ****S1**). Sensitivity, specificity, positive predictive value, and negative predictive value calculated using the SNUH-NSI, serum albumin level, NRI, and CFI in predicting in-hospital mortality are available in **Supplemental Table S6**. The SNUH-NSI demonstrated a significantly higher sensitivity than all other indices, nearly twice that of the NRI (0.82 vs. 0.45).

The results of other outcomes are summarized in **Supplemental Table S7**.

### Sensitivity analysis

Among the 4,656 patients with complete data on the SNUH-NSI parameters and a median (IQR) follow-up duration of 4.9 (2.5–7.7) years, the overall cumulative all-cause mortality rate was 10.7% (197/1,833) in the low-risk group, 23.8% (503/2,115) in the intermediate-risk group, and 36.7% (256/697) in the high-risk group. Postoperative cumulative all-cause mortality differed significantly across the three groups, with the highest and lowest rates observed in the high-risk and low-risk groups, respectively (log-rank test, pairwise comparison; all *P* < 0.001; Fig. 2b). In the multivariable analysis, both the intermediate-risk (adjusted HR, 1.55; 95% CI, 1.30–1.84; *P* < 0.001) and high-risk groups (adjusted HR, 2.65; 95% CI, 2.17–3.23; *P* < 0.001) were significant risk factors for cumulative all-cause mortality compared with the low-risk group.

## Discussion

This retrospective observational study investigated the association between preoperative malnutrition risk, assessed using the SNUH-NSI, and postoperative mortality following cardiac surgery. Our findings showed that all-cause mortality risk was 1.5- and 2.4-fold greater in patients with intermediate and high malnutrition risks, respectively. Furthermore, the model incorporating the SNUH-NSI showed better performance in predicting cardiac-cause and in-hospital mortalities than conventional nutritional indices.

The SNUH-NSI is a comprehensive screening tool for malnutrition that incorporates several previously studied parameters to assess the nutritional status of patients undergoing cardiac surgery^20^. This tool, unlike single-variable or limited-variable approaches, better reflects the complex nature of malnutrition. Although serum albumin, a traditional nutritional biomarker, has been utilized as a predictor of morbidity and mortality following cardiac surgery^21^, its sensitivity and specificity are limited. Serum albumin levels can be affected by various conditions, such as advanced liver disease, dilution caused by fluid balance, edema, nephrotic syndrome, and cancer^28,29^. Therefore, current guidelines recommend the use of serum albumin as a component of preoperative nutritional screening rather than as a standalone marker^11^. Similarly, while low BMI is an established risk factor for malnutrition and poorer postoperative outcomes^20,30^, a U-shaped relationship has been suggested by previous studies on BMI and postoperative mortality, with increased risk in obese patients^30^. In our study, the multivariable model including NRI demonstrated acceptable predictive performance; however, NRI is calculated solely by two parameters—serum albumin level and recent weight changes^24^. Furthermore, NRI cannot account for the increased risk of poor postoperative outcomes in patients who are chronically underweight or obese. Thus, the use of various multi-parameter screening tools, such as the SNUH-NSI, offers a more systemic approach to sensitively identify patients at risk for malnutrition while avoiding the risk of overfitting.

Another strength of the SNUH-NSI is its potential for the automated routine screening of hospitalized patients. Current enhanced recovery after surgery guidelines recommend routine preoperative nutritional screening^11^. However, previous multi-parameter screening tools often require exhaustive evaluations and additional human resources. Moreover, the inclusion of subjective parameters, including loss of appetite or functional impairment, necessitates prospective assessment, which hinders its suitability for routine screening in daily practice. At our institution, subjective parameters were included in the SNUH-NSI as information routinely obtained during admission. Therefore, although the SNUH-NSI comprises 11 components with subjective parameters, we were still successful in implementing an automated nutritional screening system for all inpatients, promoting a cost- and time-efficient approach^31^. As the number of risk prediction tools essential to clinical practice continues to grow, the SNUH-NSI serves as an illustrative example of an automated routine screening tool that alleviates the clinical burden on healthcare providers.

Our study highlights the effectiveness of the SNUH-NSI in identifying patients at risk of malnutrition. However, we were unable to investigate its use in nutritional interventions in this subgroup because the short time window between admission and the day of cardiac surgery prevented correction of nutritional deficiencies in the inpatient setting. While current recommendations suggest that high-risk patients should receive oral nutritional supplements for at least 7 days before major surgery^11^, this is often impractical for cardiac surgery patients due to the urgency of their condition. Furthermore, since the evidence supporting the benefits of preoperative nutritional therapy in cardiac surgery is limited, the Enhanced Recovery After Surgery Society has recommended preoperative correction of nutritional deficiencies only when feasible with a low level of evidence (class of recommendation, IIa; level of evidence, C-LD)^13^. Fortunately, small-scale randomized studies have reported that short periods (2–5 days) of preoperative nutritional support, using enteral, parenteral, or immuno-nutrition, may improve myocardial glucose metabolism and reduce nosocomial infection and hospital stay length in patients undergoing cardiac surgery^32,33^. Further large-scale randomized trials are warranted to definitively assess the impact of preoperative nutritional supplementation within the limited timeframe available in cardiac surgery patients and to determine whether delaying surgery for nutritional support would benefit selected patients. Additionally, further attention is needed to determine whether automated consultations for patients identified as high-risk for malnutrition could facilitate prompt, more comprehensive assessments, thereby enabling earlier perioperative nutritional interventions.

Despite the insights offered in this study, several limitations must be acknowledged. First, the study was retrospective in nature. Although robust adjustments were performed in the multivariable analyses to minimize bias, unmeasured confounding variables may have influenced our findings. Second, we validated the SNUH-NSI using data from a single tertiary teaching hospital with a high surgical volume^16–18^. While this may have minimized the effect of surgeon experience on postoperative outcomes, the generalizability of our findings may not be applicable to institutions with low surgical volumes. Third, given the retrospective nature of our study, we could not obtain adequate data to assess other nutritional screening tools with variables that mandate prospective data collection, such as the Malnutrition Universal Screening Tool, the Mini-Nutritional Assessment short-form, Subjective Global Assessment, Nutritional Risk Screening 2002, and the Short Nutritional Assessment Questionnaire^7^. Further prospective studies are required to directly compare the performance of these tools with the SNUH-NSI. Lastly, we did not evaluate the performance of other well-known risk prediction models, such as the European System for Cardiac Operation Risk Evaluation II or Society of Thoracic Surgeons score. Nevertheless, this aspect fell outside the scope of the current study.

## Conclusion

In this study, SNUH-NSI was found to be a good predictor of postoperative mortality in patients undergoing cardiac surgery. Preoperative malnutrition, assessed using the SNUH-NSI, was significantly associated with an increased risk of all-cause mortality following cardiac surgery. Additionally, the SNUH-NSI was superior to conventional malnutrition indices in predicting cardiac-cause and in-hospital mortalities. These findings underscore the utility of integrating the SNUH-NSI into preoperative nutritional assessments to improve risk stratification and patient outcomes.

## Electronic supplementary material

Below is the link to the electronic supplementary material.

Supplementary Material 1

## Data Availability

The datasets generated during and/or analyzed during the current study are available from the corresponding authors on reasonable request.
