# Author Correction: Performance of the comprehensive nutrition screening index in predicting mortality after cardiac surgery

**DOI:** 10.1038/s41598-025-10922-1

**Published:** 2025-07-21

**Authors:** Jaeyeon Chung, Jinyoung Bae, Seyong Park, Dong Hyouk Kim, Youn Joung Cho, Karam Nam, Yunseok Jeon, Jae-Woo Ju

**Affiliations:** 1 Department of Anesthesiology and Pain Medicine, Seoul National University Hospital, Seoul National University College of Medicine, 101 Daehak-ro, Jongno-gu, Seoul, 03080 Republic of Korea; 2 Department of Anesthesiology and Pain Medicine, Ajou University Medical Center, Ajou University School of Medicine, Suwon, Republic of Korea

Correction to: *Scientific Reports* 10.1038/s41598-024-78114-x, published online 18 November 2024

The original version of this Article omitted an affiliation for author Jinyoung Bae. The correct affiliations are listed below.

Department of Anesthesiology and Pain Medicine, Seoul National University Hospital, Seoul National University College of Medicine, Seoul, Republic of Korea

Department of Anesthesiology and Pain Medicine, Ajou University Medical Center, Ajou University School of Medicine, Suwon, Republic of Korea

The original Article has been corrected.

