# Supplementary material for: Performance of the comprehensive nutrition screening index in predicting mortality after cardiac surgery
Source: Sci Rep. 2024 Nov 18;14:28507. doi: 10.1038/s41598-024-78114-x (PMC11574065; doi:10.1038/s41598-024-78114-x)

**Supplementary Material**

**Performance of comprehensive nutrition screening index in predicting mortality after cardiac surgery**

Jaeyeon Chung**,**^a^ Jinyoung Bae,^a, 1^ Seyong Park, Dong Hyouk Kim, Youn Joung Cho, Karam Nam, Yunseok Jeon, and Jae-Woo Ju

Department of Anesthesiology and Pain Medicine, Seoul National University Hospital, Seoul National University College of Medicine, Seoul, Republic of Korea

^1^Present address: Department of Anesthesiology and Pain Medicine, Ajou University of College of Medicine, Suwon, Korea

^a^J.C. and J.B. contributed equally to this work and share the role of first author

**Corresponding author**: Jae-Woo Ju, M.D.

Clinical Assistant Professor

Department of Anesthesiology and Pain Medicine

Seoul National University Hospital, Seoul National University College of Medicine

101 Daehak-ro, Jongno-gu, Seoul 03080, Republic of Korea

Cell +82 10 4849 2489, Fax +82 2 747 8363,

E-mail: jujw701@gmail.com

**TABLE OF CONTENTS**

**Supplemental Table S1.** Univariable Cox regression analysis for all-cause mortality with individual parameters of SNUH-NSI … p. 4–5

**Supplemental Table S2.** Univariable and multivariable cox regression analysis for cardiac-cause mortality … p. 6–7

**Supplemental Table S3.** Causes of in-hospital death according to the study groups. … p. 8–9

**Supplemental Table S4.** Univariable and multivariable logistic regression analysis for in-hospital mortality … p. 10–11

**Supplemental Table S5.** Performance of logistic regression models based on different nutrition indexes for in-hospital mortality… p. 12

**Supplemental Table S6.** Sensitivity, specificity, positive predictive value, and negative predictive value nutritional indices in predicting in-hospital mortality

… p. 13

**Supplemental Table S7.** Postoperative complications and length of hospital stay according to the study groups … p. 14

**Supplemental Figure S1**. Receiver operating curve for logistic regression models with different nutrition indexes for in-hospital mortality … p. 15

**Supplemental Table S1.** Univariable Cox regression analysis for all-cause mortality with individual parameters of SNUH-NSI

| Parameters of SNUH-NSI | HR (95% CI) | *P* value |
| --- | --- | --- |
| Appetite, bad | 1.98 (1.60–2.46) | <0.001 |
| Change of weight | 1.21 (1.01–1.45) | 0.040 |
| Difficulty in digesting | 1.51 (1.22–1.86) | <0.001 |
| Diet type |  | <0.001 |
| Normal regular diet | Reference |  |
| Soft blended diet or NPO | 2.08 (1.79–2.42) | <0.001 |
| Fluid diet | 3.06 (1.64–5.70) | <0.001 |
| Serum albumin |  | <0.001 |
| ≥ 3.3 g/dL | Reference |  |
| 2.8–3.3 g/dL | 3.00 (2.52–3.57) | <0.001 |
| < 2.8 g/dL | 2.82 (2.19–3.64) | <0.001 |
| Serum cholesterol |  |  |
| ≥ 130 mg/dL | Reference |  |
| < 130 mg/dL | 1.77 (1.55–2.01) | <0.001 |
| Total lymphocyte count |  | <0.001 |
| ≥ 1500 cells/mm^3^ | Reference |  |
| 800–1500 cells/mm^3^ | 2.06 (1.81–2.35) | <0.001 |
| < 800 cells/mm^3^ | 3.91 (3.22–4.76) | <0.001 |
| Hemoglobin |  |  |
| Male ≥ 13.0 g/dL, Female ≥ 12.0 g/dL | Reference |  |
| Male < 13.0 g/dL, Female < 12.0 g/dL | 2.69 (2.37–3.05) | <0.001 |
| C–reactive protein |  |  |
| < 1 mg/dL | Reference |  |
| ≥ 1 mg/dL | 2.31 (2.02–2.64) | <0.001 |
| Body mass index |  |  |
| 18–25 kg/m^2^ | Reference |  |
| < 18 or ≥ 25 kg/m^2^ | 0.89 (0.79–1.01) | 0.065 |
| Age |  | <0.001 |
| ≤ 75 years | Reference |  |
| > 75 years | 2.93 (2.53–2.38) |  |

SNUH-NSI, Seoul National University Hospital-Nutrition Screening Index; HR, hazard ratio; CI, confidence interval; NPO, nil per os.

**Supplemental Table S2.** Univariable and multivariable cox regression analysis for cardiac-cause mortality

|  | Univariable | |  | Multivariable | |
| --- | --- | --- | --- | --- | --- |
|  | Unadjusted HR  (95% CI) | *P* value |  | Adjusted HR  (95% CI) | *P* value |
| SNUH-NSI |  | <0.001 |  |  |  |
| Low-risk | Reference |  |  | Reference |  |
| Intermediate-risk | 2.32 (1.73–3.10) | <0.001 |  | 1.39 (1.02–1.89) | 0.036 |
| High-risk | 5.24 (3.82–7.19) | <0.001 |  | 2.68 (1.91–3.77) | <0.001 |
| Age (years) | 1.06 (1.05–1.08) | <0.001 |  | 1.07 (1.05–1.08) | <0.001 |
| Male | 1.00 (0.79–1.25) | 0.969 |  |  |  |
| Smoking | 0.92 (0.68–1.26) | 0.608 |  |  |  |
| Hazardous alcohol consumption | 0.81 (0.40–1.63) | 0.546 |  |  |  |
| Comorbidities |  |  |  |  |  |
| Diabetes mellitus | 1.50 (1.18–1.90) | <0.001 |  | 1.04 (0.81–1.34) | 0.772 |
| Myocardial infarction | 1.75 (1.16–2.63) | 0.008 |  | 1.13 (0.74–1.74) | 0.573 |
| Congestive heart failure | 2.74 (2.08–3.61) | <0.001 |  | 1.31 (0.97–1.78) | 0.081 |
| Stroke/transient ischemic attack | 1.31 (0.96–1.80) | 0.090 |  | 0.99 (0.72–1.36) | 0.927 |
| Chronic obstructive pulmonary disease | 1.24 (0.51–2.99) | 0.638 |  |  |  |
| Extracardiac arteriopathy | 1.81 (1.12–2.91) | 0.015 |  | 1.54 (0.94–2.52) | 0.084 |
| Dialysis | 3.36 (2.11–5.36) | <0.001 |  | 1.17 (0.61–2.24) | 0.645 |
| Previous cardiac surgery | 1.67 (1.18–2.36) | 0.004 |  | 1.78 (1.23–2.59) | 0.002 |
| Preoperative laboratory data |  |  |  |  |  |
| Serum creatinine (mg/dl) | 1.20 (1.14–1.26) | <0.001 |  | 1.13 (1.05–1.22) | <0.001 |
| Left ventricular ejection fraction (%) | 0.96 (0.96–0.97) | <0.001 |  | 0.97 (0.96–0.98) | <0.001 |
| Type of surgery |  | 0.030 |  |  |  |
| CABG | Reference |  |  | Reference |  |
| Valve | 0.98 (0.76–1.27) | 0.892 |  | 1.38 (1.03–1.86) | 0.032 |
| Aorta | 0.42 (0.21–0.82) | 0.011 |  | 0.59 (0.29–1.18) | 0.133 |
| Valve + CABG | 0.92 (0.67–1.24) | 0.570 |  | 1.15 (0.82–1.60) | 0.419 |
| Valve + aorta | 0.37 (0.09–1.50) | 0.165 |  | 0.56 (0.14–2.29) | 0.420 |
| Year of surgery |  | 0.628 |  |  |  |
| 2008–2011 | Reference |  |  |  |  |
| 2012–2015 | 1.10 (0.84–1.43) |  |  |  |  |
| 2016–2019 | 0.95 (0.68–1.33) |  |  |  |  |
| Emergency surgery | 1.58 (1.19–2.08) | 0.001 |  | 1.10 (0.81–1.49) | 0.537 |

HR, hazard ratio; CI, confidence interval; SNUH-NSI, Seoul National University Hospital-Nutrition Screening Index; CABG, coronary artery bypass grafting surgery.

**Supplemental Table S3.** Causes of in-hospital death according to the study groups

| Cause of in-hospital death | n |
| --- | --- |
| *SNUH-NSI low risk* | *25 died out of 2158 (1.2%)* |
| Hypovolemic shock | 8 |
| Infection | 4 |
| Disseminated intravascular coagulation | 3 |
| Ischemic colitis | 3 |
| Heart failure | 2 |
| Pneumonia or ARDS | 2 |
| Renal failure | 2 |
| Brain infarct | 1 |
| *SNUH-NSI intermediate risk* | *52 died out of 2395 (2.2%)* |
| Infection | 16 |
| Pneumonia or ARDS | 14 |
| Heart failure | 7 |
| Brain infarct | 3 |
| Disseminated intravascular coagulation | 3 |
| Hypovolemic shock | 2 |
| Ischemic colitis | 2 |
| Renal failure | 2 |
| Cardiogenic shock | 1 |
| Unknown or unspecified | 2 |
| *SNUH-NSI high risk* | *60 died out of 774 (7.8%)* |
| Infection | 25 |
| Pneumonia or ARDS | 16 |
| Heat failure | 4 |
| Ischemic colitis | 4 |
| Renal failure | 3 |
| Brain infarct | 2 |
| Hypovolemic shock | 2 |
| Cardiogenic shock | 1 |
| Liver failure | 1 |
| Rhabdomyolysis | 1 |
| Unknown or unspecified | 1 |

ARDS, acute respiratory distress syndrome.

**Supplemental Table S4.** Univariable and multivariable logistic regression analysis for in-hospital mortality

|  | Univariable | |  | Multivariable | |
| --- | --- | --- | --- | --- | --- |
|  | Unadjusted OR  (95% CI) | *P* value |  | Adjusted OR  (95% CI) | *P* value |
| SNUH-NSI |  | <0.001 |  |  |  |
| Low-risk | Reference |  |  | Reference |  |
| Intermediate-risk | 1.89 (1.17–3.06) | 0.009 |  | 1.19 (0.71–1.97) | 0.510 |
| High-risk | 7.17 (4.46–11.52) | < 0.001 |  | 3.14 (1.87–5.26) | < 0.001 |
| Age (years) | 1.04 (1.03–1.06) | < 0.001 |  | 1.05 (1.03–1.07) | < 0.001 |
| Male | 0.92 (0.65–1.29) | 0.619 |  |  |  |
| Smoking | 1.05 (0.66–1.67) | 0.827 |  |  |  |
| Hazardous alcohol consumption | 0.68 (0.22–2.17) | 0.537 |  |  |  |
| Comorbidities |  |  |  |  |  |
| Diabetes mellitus | 1.35 (0.94–1.94) | 0.105 |  |  |  |
| Myocardial infarction | 1.35 (0.70–2.59) | 0.375 |  |  |  |
| Congestive heart failure | 2.76 (1.81–4.21) | < 0.001 |  | 1.24 (0.76–2.02) | 0.395 |
| Stroke/transient ischemic attack | 1.13 (0.71–1.81) | 0.608 |  |  |  |
| Chronic obstructive pulmonary disease | 2.20 (0.88–5.50) | 0.093 |  | 1.50 (0.55–4.06) | 0.428 |
| Extracardiac arteriopathy | 1.22 (0.53–2.80) | 0.641 |  |  |  |
| Dialysis | 7.45 (4.57–12.13) | < 0.001 |  | 3.01 (1.34–6.77) | 0.008 |
| Previous cardiac surgery | 1.50 (0.87–2.58) | 0.148 |  |  |  |
| Preoperative laboratory data |  |  |  |  |  |
| Serum creatinine (mg/dl) | 1.29 (1.21–1.38) | < 0.001 |  | 1.14 (1.03–1.28) | 0.016 |
| Left ventricular ejection fraction (%) | 0.97 (0.96–0.98) | < 0.001 |  | 0.98 (0.96–0.99) | 0.002 |
| Type of surgery |  | <0.001 |  |  |  |
| CABG | Reference |  |  | Reference |  |
| Valve | 2.07 (1.30–3.30) | 0.002 |  | 3.58 (2.14–6.01) | < 0.001 |
| Aorta | 4.12 (2.27–7.47) | <0.001 |  | 5.86 (3.05–11.27) | <0.001 |
| Valve + CABG | 2.34 (1.39–3.95) | <0.001 |  | 2.73 (1.55–4.78) | <0.001 |
| Valve + aorta | 0.82 (0.19–3.48) | 0.787 |  | 3.15 (0.67–14.69) | 0.145 |
| Year of surgery |  | <0.001 |  |  |  |
| 2008–2011 | Reference |  |  | Reference |  |
| 2012–2015 | 0.86 (0.60–1.25) | 0.443 |  | 0.68 (0.46–1.01) | 0.054 |
| 2016–2019 | 0.30 (0.18–0.50) | <0.001 |  | 0.25 (0.15–0.44) | <0.001 |
| Emergency surgery | 3.07 (2.11–4.47) | < 0.001 |  | 1.73 (1.12–2.66) | 0.013 |

OR, odds ratio; CI, confidence interval; SNUH-NSI, Seoul National University Hospital-Nutrition Screening Index; CABG, coronary artery bypass grafting surgery.

**Supplemental Table S5.** Performance of logistic regression models based on different nutrition indexes for in-hospital mortality

| **Nutrition index** |  | AUC  (95% CI) | AIC | McFadden’s  pseudo R^2^ |
| --- | --- | --- | --- | --- |
| SNUH-NSI | Low vs. Intermediate vs. High | 0.831 (0.804–0.866) | 1082.5 | 0.175 |
| Serum albumin | <2.5g/dL vs. ≥2.5g/dL | 0.813 (0.783–0.851) | 1102.6 | 0.158 |
|  | <3.0g/dL vs. ≥3.0g/dL | 0.819 (0.792–0.857) | 1098.3 | 0.161 |
| BMI WHO (kg/m^2^) | <18.5 vs 18.5≤ BMI <25 vs.  25≤ BMI <30 vs.  ≥30 | 0.822 (0.794–0.859) | 1102.9 | 0.161 |
| BMI Asia (kg/m^2^) | <18.5 vs. 18.5≤ BMI <23 vs. 23≤ BMI <25 vs. ≥25 | 0.818 (0.792–0.856) | 1106.1 | 0.158 |
| Nutritional Risk Index | >100 (no risk) vs.  97.5≤ NRI <100 (mild) vs.  83.5≤ NRI <97.5 (intermediate) vs.  ≤83.5 (severe) | 0.816 (0.788–0.856) | 1106.5 | 0.158 |
| Chart-derived Frailty Index | 0–2 (low risk) vs.  3–5 (high risk) | 0.825 (0.794–0.862) | 1088.2 | 0.169 |
| Model without Nutrition index | – | 0.812 (0.782–0.850) | 1106.0 | 0.154 |

AUC, area under the receiver operating curve; CI, confidence interval; AIC, Akaike information criterion; SNUH-NSI, Seoul National University Hospital-Nutrition Screening Index; BMI, Body mass index; WHO, World Health Organization classification; Asia, Asia-Pacific classification.

**Supplemental Table S6.** Sensitivity, specificity, positive predictive value, and negative predictive value nutritional indices in predicting in-hospital mortality

| **Nutrition index** |  | Sensitivity | Specificity | Positive predictive value | Negative predictive value |
| --- | --- | --- | --- | --- | --- |
| SNUH-NSI | low risk vs.  intermediate or high risk | 0.82 | 0.41 | 0.04 | 0.99 |
| Serum albumin | <2.5g/dL vs. ≥2.5g/dL | 0.06 | 0.99 | 0.15 | 0.98 |
|  | <3.0g/dL vs. ≥3.0g/dL | 0.18 | 0.96 | 0.10 | 0.98 |
| Nutritional Risk Index | >100 (no risk) vs.  ≤100 (mild or higher risk) | 0.45 | 0.76 | 0.05 | 0.98 |
| Chart-derived Frailty Index | 0–2 (low risk) vs.  3–5 (high risk) | 0.36 | 0.93 | 0.12 | 0.98 |

**Supplemental Table S7**. Postoperative complications and length of hospital stay according to the study groups

|  | SNUH-NSI | | |  |
| --- | --- | --- | --- | --- |
|  | Low risk  (n=2,158) | Intermediate risk  (n=2,395) | High risk  (n=774) | *P* value |
| Acute kidney injury | 475 (22.0) | 870 (36.3) | 379 (49.0) | <0.001 |
| Newly initiated renal replacement therapy | 4 (0.2) | 18 (0.8) | 15 (1.9) | <0.001 |
| Prolonged intubation ≥ 48 hours | 236 (10.9) | 401 (16.7) | 252 (32.6) | <0.001 |
| Length of ICU stay (days) | 2 (1–3) | 3 (1–5) | 4 (2–9) | <0.001 |
| Length of hospital stay (days) | 9 (7–13) | 11 (8–18) | 17 (10–32) | <0.001 |
| Readmission within 90 days | 224 (10.4) | 393 (16.4) | 137 (17.7) | <0.001 |

Values are expressed as the median (interquartile range) or number (proportion).

SNUH-NSI, Seoul National University Hospital-Nutrition Screening Index; ICU, intensive care unit.

**Supplemental Figure S1.** Receiver operating curve for logistic regression models with different nutrition indexes for in-hospital mortality. Only models with SNUH-NSI, NRI, CFI and without nutrition index are displayed. Alb 2.5 and 3.0 indicate model with serum albumin cut-off of 2.5mg/dL and 3.0mg/dL.

AUC, area under the receiver operating curve; CI, confidence interval; SNUH-NSI, Seoul National University Hospital-Nutrition Screening Index; BMI, Body mass index; WHO, World Health Organization classification; Asia, Asia-Pacific classification; NRI, Nutritional Risk Index; CFI, Chart-derived Frailty Index.


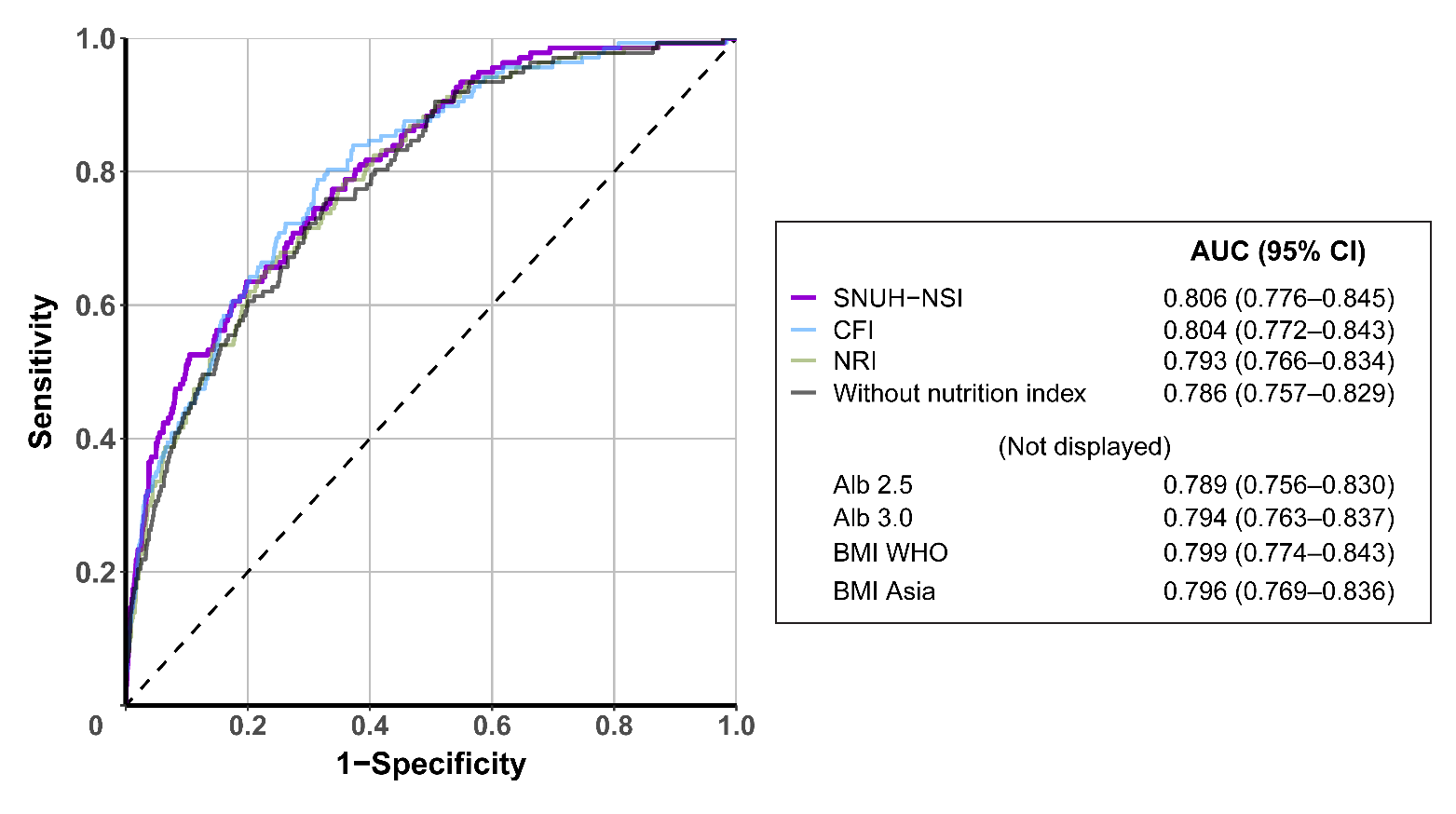

Supplement: Supplementary file 1 — Supplementary Material 1 [file 41598_2024_78114_MOESM1_ESM.docx]
